# Roe Deer *Capreolus capreolus* Home Range Size and Daily Distance in the Agricultural Landscape of East‐Central Poland in Relation to Natural and Anthropogenic Factors

**DOI:** 10.1002/ece3.74550

**Published:** 2026-10-28

**Authors:** Daniel Klich, Michalina Gmaj, Wojciech Bielecki, Maria Sobczuk, Łukasz Mrugała, Paweł Nasiadka, Marta Gałązka, Marcin Świątek

**Affiliations:** ^1^ Department of Animal Genetics and Conservation, Faculty of Bioeconomy and Food Systems Warsaw University of Life Sciences (SGGW) Warsaw Poland; ^2^ Department of Pathology and Veterinary Diagnostics, Faculty of Veterinary Medicine Warsaw University of Life Sciences (SGGW) Warsaw Poland; ^3^ Lasy Miejskie Warszawa Warsaw Poland; ^4^ Department of Animal Breeding and Nutrition, Faculty of Bioeconomy and Food Systems Warsaw University of Life Sciences (SGGW) Warsaw Poland

**Keywords:** core area, home range, Poland, roe deer, wind farm

## Abstract

Home range and daily distance traveled are the basic parameters of animal habitat utilization and emerge from complex interactions between animals and their environment. In this study, the home ranges, core areas, and daily distances traveled of 10 collared roe deer in east‐central Poland were analyzed. The mean home range size for all animals was 136.1 ha (minimum convex polygon of 95% of points), and 69 ha (95% kernel density estimation); the mean core area was 10.9 ha (50% kernel density estimation). Core areas were larger in males than in females and larger in the winter period than in the growing period. Home range size decreased with increasing forest cover and increased with the number of wind turbines. Core area size was influenced only by the sex of the animal and forest cover. Neither the size of the home range nor the size of the core area depended on road density or settlement cover. The relationship between home range size and sex, natural factors, including forest cover, and season, is consistent with previous studies. Mean daily distance traveled for all animals was 3.14 km, and was lower in the growing period than in winter, and in females than in males. The daily distance traveled also decreased with increasing forest cover. Our study indicated a new anthropogenic factor shaping roe deer's spatial behavior in agricultural landscapes (wind farms). We concluded that roe deer have probably adapted behaviorally to wind farms as a new landscape feature. They probably treat wind farms like human settlements, generally avoid them, and use habitats at an appropriate distance from the turbines. We cannot, however, exclude the occasional fleeing behavior of roe deer near turbines. Further research should include a more detailed assessment of the behavior of roe deer and other terrestrial mammal species under the influence of wind farms.

## Graphical abstract

The home ranges, core areas, and daily distances traveled of 10 collared roe deer in the agricultural landscape of east‐central Poland were analyzed. We demonstrated the dependence of the home range size and daily distance on natural and anthropogenic factors. We first demonstrated a relationship between home range size and the wind farm impacts on roe deer.

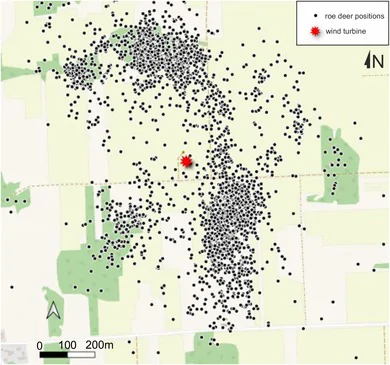

## 1 Introduction

Habitat utilization is a fundamental component of animal ecology, reflecting how individuals interact with their environment to obtain resources necessary for survival and reproduction (Boitani and Fuller 2000). Most resident mammals restrict their movements to relatively well‐defined areas rather than moving randomly, a concept described as the home range (Burt 1943). Understanding patterns of activity, home range size, and spatial behavior is therefore essential for interpreting ecological strategies and responses to environmental conditions (Börger et al. 2008; Nathan et al. 2008; Spencer 2012). Home ranges emerge from complex interactions between individuals and their environment, reflecting both the spatial distribution of resources and behavioral decisions shaped by natural selection (Mitchell and Powell 2004; Powell and Mitchell 2012). Consequently, home range behavior can be viewed as an interplay between environmental conditions and an animal's understanding of that environment, its cognitive map, which guides movement decisions and resource use (Börger et al. 2008; Gehr et al. 2020; Spencer 2012). Linking observed space‐use patterns with the cognitive processes underlying movement may therefore improve our ability to interpret animal home ranges (Powell and Mitchell 2012).

The roe deer ( *Capreolus capreolus* ) is a widespread ungulate species across Europe and can serve as a valuable model for studying spatial ecology in human‐modified landscapes (Ionescu et al. 2025; Lorenzini et al. 2022). Originally associated with early successional forest habitats, roe deer now occupy a broad range of environments, including agricultural landscapes and fragmented habitats, demonstrating considerable behavioral and ecological plasticity (Hewison et al. 2001, 2009; Jepsen and Topping 2004). Home range size and spatial behavior in roe deer are influenced by numerous factors, including habitat composition, availability of woodland patches, and landscape structure (Bonnot et al. 2013; Cargnelutti et al. 2002; Hewison et al. 2001; Lovari et al. 2017; Marchal et al. 1998; Padié et al. 2015; Saïd and Servanty 2005). Home range size is also influenced by intrinsic factors, including sex, age, population density, and reproductive status (José and Lovari 1998; Kjellander et al. 2004; Richard et al. 2008; Saïd et al. 2009; Tufto et al. 1996), as well as by perceived risk associated with predators, recreational activities, hunting pressure, and other anthropogenic disturbances, including seasonal variation in human activity (Benhaiem et al. 2008; Gehr et al. 2026; Kjøstvedt et al. 1998; Mysterud et al. 1999; Mols et al. 2022; Guillet et al. 1996). As a consequence, individuals may adjust their spatial behavior and home range size in response to environmental conditions and risk perception. In addition to home range size, movement‐related metrics such as daily distance traveled may provide further insight into how individuals adjust their movement behavior in response to landscape features and anthropogenic disturbances (Stache et al. 2013).

Human activities increasingly shape wildlife habitats by altering resource distribution, landscape structure, and disturbance regimes (McIntyre and Hobbs 1999; Turner 2010). Habitat fragmentation, infrastructure development, and human presence can modify animal movement patterns and space use by increasing perceived risk or by physically changing the landscape (Benhaiem et al. 2008; Fahrig 2007). Among rapidly expanding forms of human infrastructure are wind farms, which are becoming prominent features in many landscapes due to the growing demand for renewable energy (Beiter et al. 2022; Bošnjaković et al. 2022). Besides the turbines themselves, associated infrastructure such as access roads and maintenance activities may increase human presence and habitat fragmentation, potentially influencing wildlife behavior and spatial ecology (Helldin et al. 2012; Lovich and Ennen 2011; Qin et al. 2025; Schöll and Nopp‐Mayr 2021).

Given the potential influence of human infrastructure on roe deer spatial behavior and the limited evidence on the effects of wind farms on terrestrial mammals, this study aimed to assess the impact of natural and anthropogenic factors on home range size, core area size and location, and daily distance traveled in a human‐modified landscape. Existing studies provide evidence on the response of roe deer to wind farm infrastructure, with some reporting reduced activity, changes in spatial distribution, or elevated stress levels (Kim et al. 2025; Klich et al. 2020, 2024; Łopucki et al. 2017), while others found minimal or no effects on occupancy (Kim et al. 2023). Wind farms may indirectly influence roe deer by modifying the landscape and its associated spatial distribution, rather than through direct effects of the turbines themselves (Gmaj and Klich 2026).

By linking observed space‐use patterns to landscape features, our study sheds light on the potential effects of human infrastructure on the spatial ecology of a widespread ungulate.

## 2 Methods

The study was conducted in east‐central Poland in agricultural landscapes with forest patches and settlements. The study area covered three wind farms and their surroundings: two in Węgrów: Korytnica I (52°23′6″ N, 21°46′14″ E) and Korytnica II (52°20′50″ N, 21°53′60″ E), and one in Rawa Mazowiecka (51°49′5″ N, 20° 3′3″ E) (Figure 1). In the Rawa Mazowiecka study area, a total of 57 wind turbines were present, of which 47 were constructed between 2012 and 2017, and an additional 10 were installed in 2024. In Węgrów (Korytnica I and Korytnica II), there were 39 turbines (25 built in 2015 and 14 added in 2022–2023). This area is characterized by average seasonal temperatures of −2.3°C in winter and 17.4°C in summer, with a clear upward trend over time, especially during winter and spring (Marosz et al. 2011). Hunting activity across the study sites was similar. Hunting activity can significantly influence roe deer movement, habitat utilization, and vigilance (e.g., Benhaiem et al. 2008; Bonnot et al. 2013; Sönnichsen et al. 2013; Picardi et al. 2019). This is important because in recent years, due to ASF, intensive hunting was conducted in some regions of Poland to reduce wild boar populations (e.g., Nasiadka et al. 2025). However, during the years of research in the studied areas, hunting was conducted according to a typical hunting plan. In all areas, wolves were the only large predators. However, their presence was sporadic. No detailed data on wolf activity were recorded. No unusual or intensive human activity was observed in any of the study areas.

**FIGURE 1:**
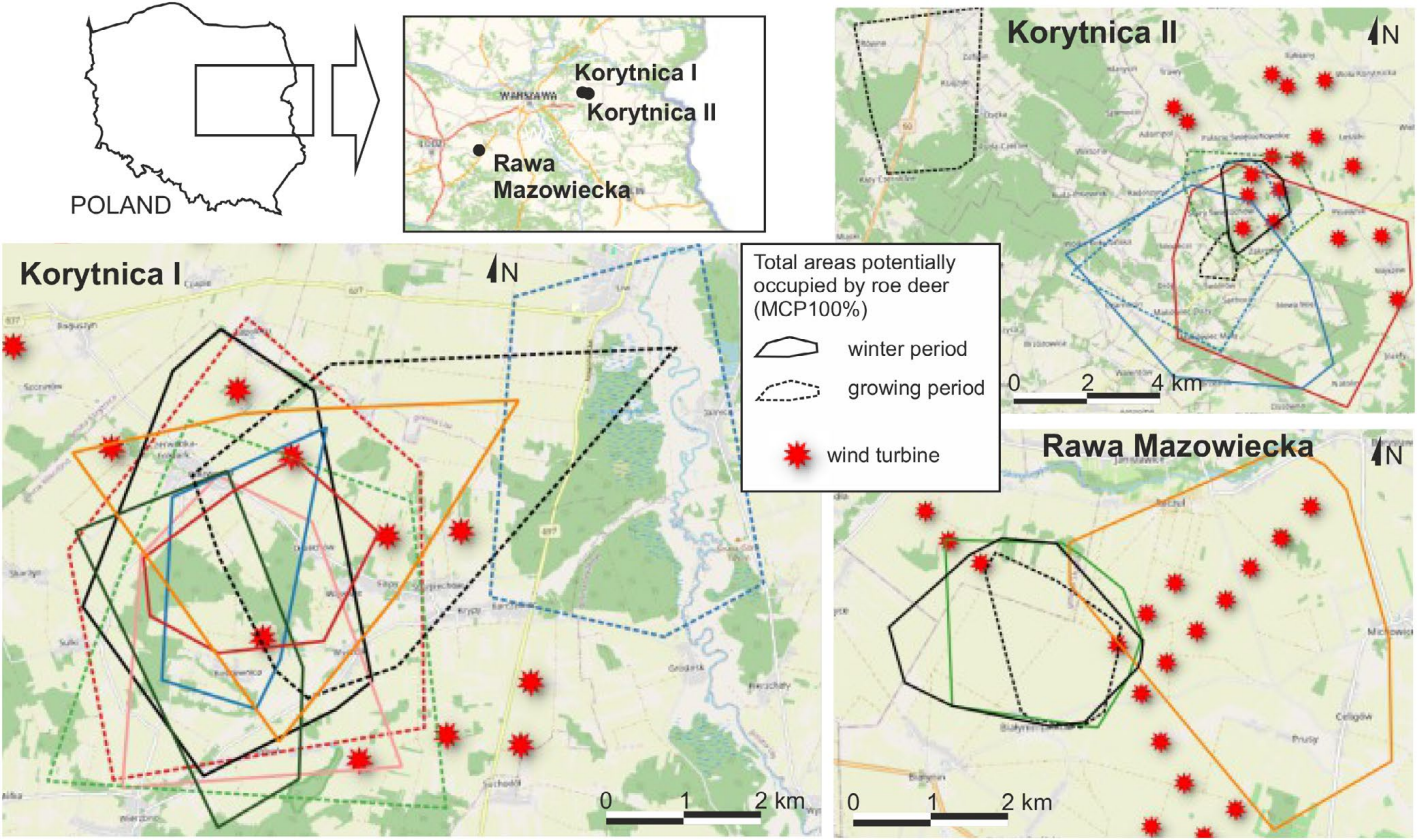
Location of the study sites and roe deer space‐use areas derived from GPS telemetry data. Polygons represent total areas potentially occupied by roe deer, estimated using the 100% minimum convex polygon (MCP) method for the winter and growing periods.

We collected telemetry data from 10 adult roe deer (seven females and three males) between 2023 and 2026 (three individuals in the Korytnica I, four individuals in the Korytnica II, and three individuals in the Rawa Mazowiecka). Detailed information on the collar operating periods has been presented in Table 1. The roe deer were captured using nets and pharmacological immobilization and equipped with GPS‐GSM transmitters (Impala collars by Ecotone, Poland). At that time, we estimated the age of the animals based on tooth wear. We used nets with variable mesh sizes ranging from 10 to 30 cm, up to 2.5 m high, and a total length of 800 m. The capture was assisted by local hunters and volunteers. Pharmacological immobilization involved a combination of drugs: medetomidine, ketamine, and butorphanol. A veterinarian was always present during the capture process, assisting with collaring and monitoring the deer's health and release. The process was carried out in accordance with the permits issued by the Ministry of the Environment (DLŁ‐WŁ.8142.19.2022.ABR, DLŁ‐WŁ.8142.7.2024.ABR) and the Local Ethics Committee (WAW2/109/2022, WAW2/003/2024). Field activities were carried out in agreement, and with the assistance of local hunting clubs leasing hunting grounds, where attempts were made to capture roe deer. The collars were set to 1 fix per hour, but one collar later scored significantly fewer points per day, which was probably the result of disruptions in the module's operation.

**TABLE 1:** Period of GPS data acquisition for a given collared individual.

ID	Sex	Age (years)	Period	Study site
SARN1	M	3	02.02.23–08.04.24	Korytnica II
SARN2	M	2	01.02.23–18.08.24	Korytnica II
SARN3	F	3	03.02.23–12.10.23	Korytnica II
SARN4	F	4	09.02.23–26.10.23	Korytnica II
SARN5	F	6	01.03.23–25.04.24	Korytnica I
SARN6	F	2	01.03.23–30.09.24	Korytnica I
SARN7	F	2	28.02.23–14.03.24	Korytnica I
SARN17	F	3	25.01.25–23.06.25	Rawa Mazowiecka
SARN21	M	5	11.02.25–30.06.25	Rawa Mazowiecka
SARN18	F	3	21.11.25–31.01.26	Rawa Mazowiecka

### 2.1 Data Elaboration and Statistics

Data collected from all individuals were analyzed to exclude false positives. Manual detection of outliers was carried out by three people independently. The data were then divided into two periods: the growing period (April to September, when individuals typically reside alone) and the winter period (October to March, when individuals typically reside in groups). In the case of two females, periods of longer distance movements (approx. 6 and 10 km) between the winter and summer home ranges were excluded. If the collar's operational time extended beyond two consecutive periods (winter and growing), the subsequent third period was treated separately, allowing an individual to yield more than two periods. In total, the data were divided into 21 periods (of which 9 were for the growing period and 12 were for the winter period, as well as 7 for males and 14 for females). For each period, the following were delimited in QGIS 3.4: (a) MCP100 (Minimum Convex Polygon of all location points)—the total area potentially occupied by an individual in given period, (b) MCP95 (Minimum Convex Polygon of 95% location points)—the home range of the potential habitat utilization area (HR), (c) KDE95 (Kernel Density Estimation of 95% location points)—the typical home range (HR), (d) KDE50 (Kernel Density Estimation of 50% location points)—the typical core area of home range (CR). The area of each of the above metrics was calculated. Additionally, centroids were determined for the core areas (KDE50), which represented the locations of the core areas. If there was more than one core area polygon for a given individual in a given period, the centroid was determined for each of them separately. A total of 34 centroids were obtained for core areas.

We also calculated the minimum daily distances traveled (DD) for each individual in the given period. For this purpose, we used the ‘geosphere’ package in R. The calculation of daily distance traveled was based on summing the distances between consecutive locations of a given individual within a 24‐h. We excluded one summer period from these calculations, in which a significantly lower number of fixes per day was recorded. In total, we calculated the mean DD for 20 periods.

For further analyses, vector layers of landscape elements in the study sites were acquired or created. We acquired vector data for three land cover types (forests, settlements, and roads) from the Topographic Objects Database (BDOT10k) on Geoportal (https://www.geoportal.gov.pl). All landscape elements were verified on the ground, and if necessary, corrections were made to individual ground cover layers. We also created a point layer of wind turbines in the study sites. All turbines were first marked in Google Earth and verified on the ground, and additional turbines were added that were not yet present on the map.

Next, we calculated the ground cover layers in the individual's surroundings. We considered MCP100 as representing the surrounding area. Within this area, we estimated the proportion of two land‐cover types: forest and settlements. Road density was expressed as meters per ha. Additionally, we counted the number of wind turbines within the MCP100 area as a measure of the roe deer's exposure to wind energy infrastructure. The number of turbines indicated the potential impact of this infrastructure on the roe deer. We also verified the inverse relationship that could result from the dependence of the number of turbines on the MCP100 area and the correlation of MCP100 area with the other HR/CR metrics. In such a case, higher HR/CR would result in a larger MCP100 area and, consequently, more turbines would have been included in the analyses. However, there was no correlation of MCP100 with the number of turbines (*r* = 0.332, *p* = 0.141), with MCP95 (*r* = 0.375, *p* = 0.094), with KDE95 (*r* = −0.114, *p* = 0.624), and with KDE50 (*r* = −0.170, *p* = 0.462). We also calculated the distance of each core area from landscape elements (forests, settlements, roads, and wind turbines) based on their centroids. Map elaboration was performed in QGIS 3.14.

The statistical analyses consisted of assessing the influence of landscape elements on HR size (expressed as MCP95 and KDE95) and core area size (KDE50), comparing the distances of core areas to landscape elements, and assessing the influence of landscape elements on minimal daily distance traveled. Due to the limited sample size, a preliminary selection of explanatory variables was made. For this purpose, a Pearson correlation matrix was created for forest cover, settlement cover, road density, and the number of turbines with the size of MCP95, KDE95, and KDE50. If a correlation occurred in even one of the HR metrics, or if it was close to significance, that variable was included in the analyses. We also checked whether the explanatory variables showed too high a correlation with each other. If a high correlation (*r* ≥ |0.7|) was found, one of the variables would be excluded from the analyses. However, no such relationship was found between the explanatory variables. The correlation matrix showed that significant correlations with HR metrics occurred only for forest cover and the number of turbines (Figure 2). These two variables (as covariates) were used in further analyses. Before selecting the model, we also checked for normality using the Shapiro–Wilk test. Since the dependent variable did not exhibit a normal distribution, a generalized linear model with a gamma distribution was used. In the model (similar for each HR metric), we used four variables: settlements, turbines, period, and sex of animals. We did not perform model selection because we wanted to include the same variables in individual models for different HR metrics to compare their impact. We only verified the model quality with the null model based on AIC values.

**FIGURE 2:**
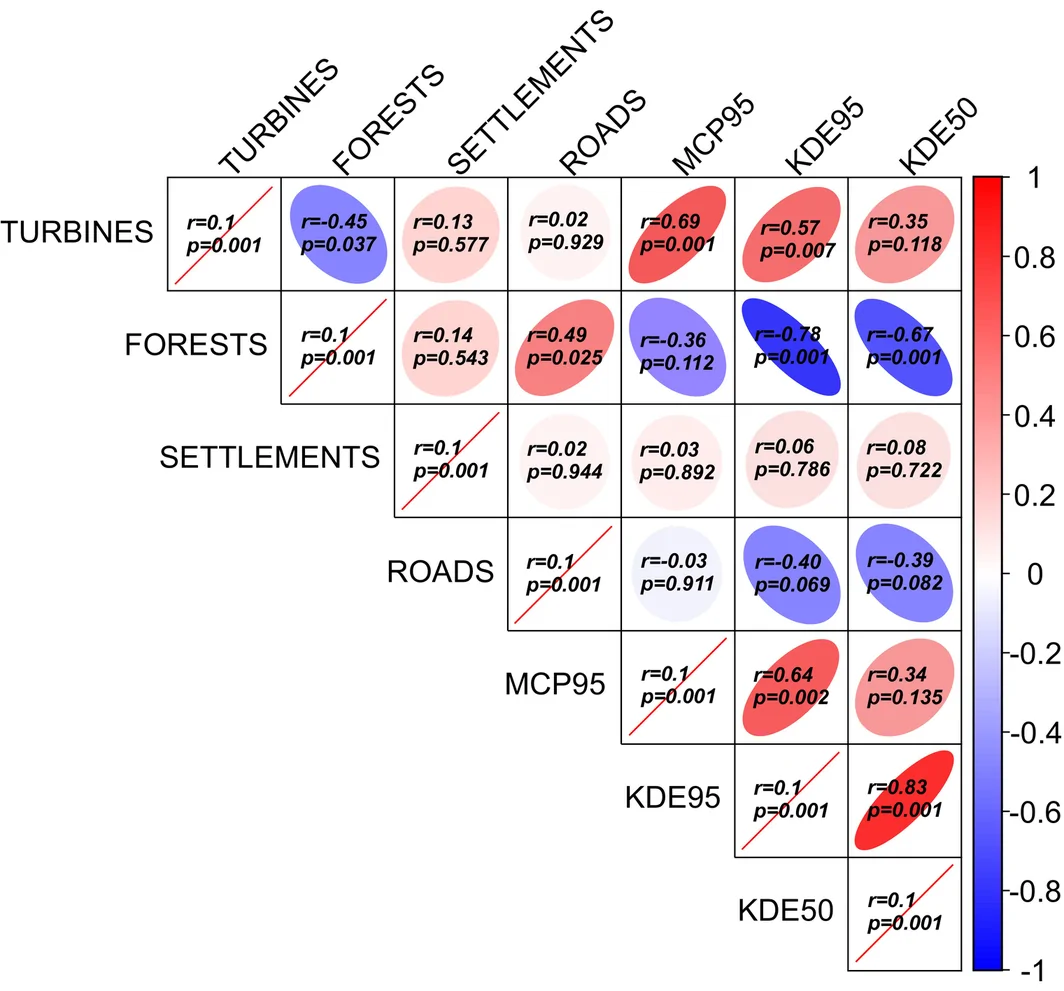
Pearson's correlation coefficients for HR/CA (home range and core area) metrics and landscape variables, which served as potential explanatory variables in further analyses (for details, see description in methods). The color saturation and the shape of the oval represent the strength of the correlation (the more saturated the color and the shape closer to a line, the stronger the correlation).

We compared the distances of core areas from landscape elements only for the given period for the given animal with wind turbines in MCP100. We therefore excluded four records, leaving a total of 30 centroids for comparison. Because the distance measurements from landscape elements were for the same core areas, we performed statistical analysis using Friedman's test for multiple dependent variables. We assumed that the distances of a given core area from a given landscape element depended on the distances of that core area from other landscape elements.

Statistical analysis of the mean minimum daily distance traveled was performed similarly to HR metrics. Correlations between potential explanatory variables were examined, and covariates were selected for the model based on this analysis. Only forest cover showed a significant correlation with DD, so it was the only linear variable in the model. Two categorical variables were also added for comparison purposes: period and sex of animal. The Shapiro–Wilk test indicated that the dependent variable (DD) was normally distributed, so the general linear model was used.

## 3 Results

The mean size of the KDE95 area was 69 ha (Table 2). Higher values that is, 81.3 ha were recorded during winter However, during the growing period, this area was much lower and equaled 52.6 ha. Males also showed higher KDE95 size values compared to females. MCP95 was on average more than twice the size of the home range (KDE95), averaging 180 ha. Similar relationships of HR sizes were found for seasons, but in contrast, females presented higher MCP95 than males. The core area (KDE50) was less than 16% of the home range KDE95 and was 10.9 ha. The core area of males was approximately 20% larger than that of females, and similarly, in the winter period, it was larger than in the growing period.

**TABLE 2:** Mean (±SE) values of home range size (HR expressed by MCP95, KDE95) and core area (CA expressed by KDE50) of roe deer with regard to sex of animals and period of the year.

Group		HR‐MCP95 (ha)	HR‐KDE95 (ha)	CA‐KDE50 (ha)	*N*
Sex	Females	144.4 ± 46.2	66.3 ± 17.7	10.2 ± 2.7	14
	Males	119.7 ± 14.4	74.5 ± 28.1	12.3 ± 4.6	7
Period	Growing	77.0 ± 9.6	52.6 ± 5.9	9.9 ± 1.3	9
	Winter	180.5 ± 66.0	81.3 ± 11.9	11.6 ± 1.6	12
Total		136.1 ± 30.9	69.0 ± 6.7	10.9 ± 0.98	21

HR size in both MCP95 and KDE95 was significantly dependent on forest cover and the number of wind turbines in the surroundings (Table 3). Both models were significant and showed much lower AIC values than the null models (chi^2^ = 32.122, *p* < 0.001 for MCP95; chi2 = 43.258, *p* < 0.001 for KDE95). However, the impact of these landscape elements differed: while an increase in the number of wind turbines increased HR size, an increase in forest cover decreased HR size (Figure 3). HR size for both metrics was significantly smaller during the growing season, but only in females was it significantly smaller for KDE95. The model for core area size (KDE50) was also significant, with a much lower AIC than the null model (chi^2^ = 19.836, *p* < 0.001). Core area size presented similar relations with explanatory variables, but the differences were significant only for forest cover and sex. Period and turbines showed trends similar to those for HR, but they were not significant (Table 3).

**TABLE 3:** Effect of forest cover (Forest Cover), number of turbines (Turbines), season (Period), and sex on roe deer home range (HR expressed by MCP95 and KDE95) and core area (CA expressed by KDE50) size (winter period and males act as reference categories). Effects obtained in a generalized linear model with a gamma distribution, where each HR/CA metric was a dependent variable in a separate model. Only the sex of animals did not significantly influence the home range size expressed by MCP95%. All variables were significant in the model of home range size, as expressed by KDE95%. Only the forest cover and sex of animals significantly influenced the core area size (KDE50%).

Method	Source	*B*	*St. Error*	*Wald χ* ^*2*^	*p*
HR MCP95	Intercept	4.854	0.265	335.511	< 0.001*
	Forest Cover	−1.963	0.767	6.505	0.011*
	Turbines	0.192	0.037	26.860	< 0.001*
	Period (growing)	−0.436	0.182	5.753	0.016*
	Sex (F)	−0.264	0.185	2.038	0.153
HR KDE95	Intercept	4.763	0.118	1639.072	< 0.001*
	Forest Cover	−2.531	0.349	52.592	< 0.001*
	Turbines	0.059	0.017	12.752	< 0.001*
	Period (growing)	−0.279	0.074	14.277	< 0.001*
	Sex (F)	−0.246	0.078	10.073	0.002*
CA KDE50	Intercept	2.942	0.186	251.085	< 0.001*
	Forest Cover	−2.362	0.556	18.036	< 0.001*
	Turbines	0.030	0.026	1.344	0.246
	Period (growing)	−0.035	0.114	0.097	0.755
	Sex (F)	−0.300	0.120	6.271	0.012*

*Note:* Significant differences were marked with an asterisk (*).

**FIGURE 3:**
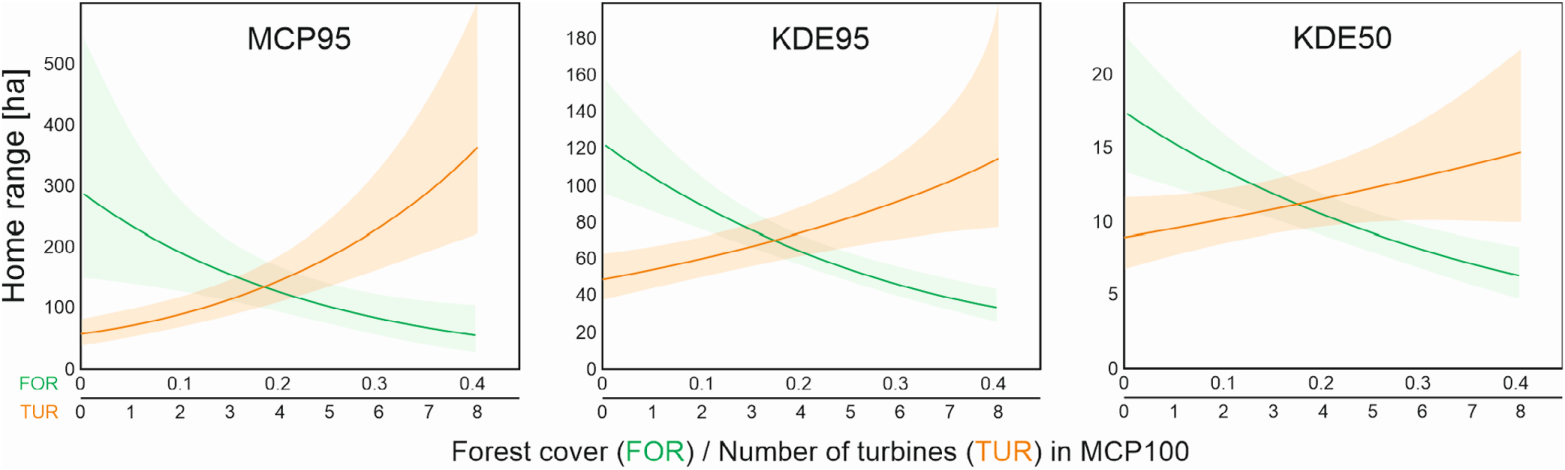
Relationship of the roe deer home range size (MCP95 and KDE95) and core area (KDE50) with the number of turbines (orange) and forest cover (green). The solid lines show the predicted HR/CA sizes, and the colored areas indicate the confidence intervals. Relations were estimated using a generalized linear model with a gamma distribution, with each HR/CA metric as a dependent variable in a separate model. Home range and core area size significantly decrease with an increase in the forest cover; home range increased significantly with the increase in turbine numbers.

Friedman's test revealed significant differences in the distances of the core area from landscape elements (test statistic = 35.320, *p* < 0.001). The median distance from forests, despite significant inter‐individual variation, was the lowest (Me = 49.8 m) (Figure 4). It was significantly lower than the distance from settlements (Me = 680.3 m) or wind turbines (Me = 854.3 m). Roe deer intensively used areas quite close to roads (Me = 154.1 m). This distance was significantly shorter than for wind turbines and settlements.

**FIGURE 4:**
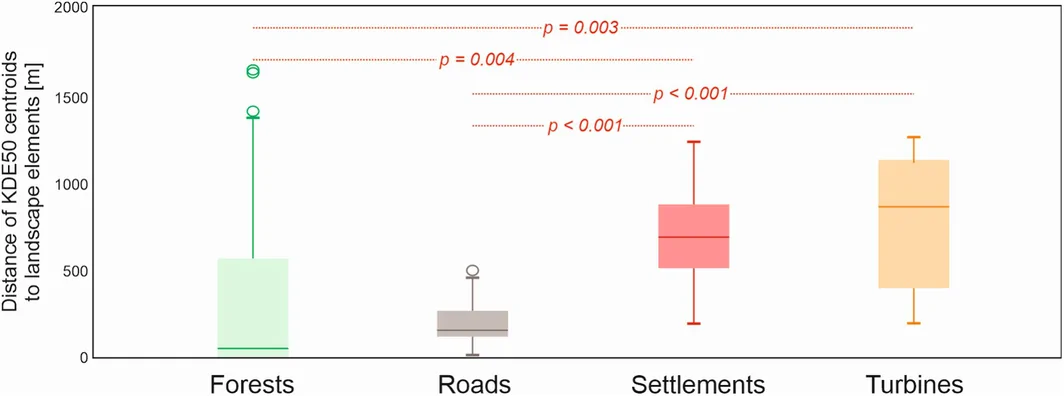
Boxplot (ME, Q1–Q3, and outliers) of distances of core areas (CA) centroids from landscape elements and pairwise comparisons in Friedman's test. The distance from core areas to settlements did not differ statistically from the distance to turbines; also, the distance from core areas to forests did not differ statistically from the distance to roads.

The mean DD for all individuals was 3.14 km (±0.12 SE). The mean DD for females was 2.96 km (±0.13 SE), and for males was about 17% higher at 3.47 km (±0.19 SE). The mean daily distance traveled in winter was 3.44 km (±0.17 SE), which was about 28% higher than in summer at 2.69 km (±0.07 SE). The model for daily distance traveled (DD) was significant and explained a fairly high percentage of DD variability (*F* = 18.630, *p* < 0.001, *R* ^2^ = 0.78). All three explanatory variables were significant (Table 4). Mean DD decreased significantly with increasing forest cover. It was also significantly lower during the growing period than during winter. Females also had significantly lower DD than males, but this difference was less pronounced than between periods (Table 4).

**TABLE 4:** Effect of Forest cover, season (Period), and sex on the mean minimal daily distance traveled (DD) of roe deer (winter period and males act as reference categories). Effects obtained in a general linear model, where DD played a dependent variable. All presented factors significantly influenced the daily distance traveled by roe deer.

Source	*B*	*St. Error*	*t*	*p*
Intercept	4.046	0.157	25.765	< 0.001*
Forest Cover	−1.934	0.564	−3.430	0.003*
Period (growing)	−0.642	0.129	−4.993	< 0.001*
Sex (F)	−0.411	0.132	−3.124	0.007*

*Note:* Significant differences were marked with an asterisk (*).

## 4 Discussion

Our study is the first to utilize modern telemetry in European roe deer research in Poland. Previous studies conducted in the 20th century relied on tagging and observing individuals or on radiotelemetry (Fruziński et al. 1983; Wasilewski 2001). Ours is therefore the first to provide information on roe deer home ranges and core areas in east‐central Poland, as well as daily distances traveled based on high‐quality telemetry data.

Despite using different methods, the estimated HR sizes can be quite similar. The HR value based on MCP95 for roe deer in open areas of southern France (using radiotelemetry), averaged 108 ha during the growing season and approximately 200 ha during the winter (Cargnelutti et al. 2002). These values are slightly higher but comparable to those we obtained in east‐central Poland, even if radiotelemetry, although it can provide data unavailable with GPS telemetry, usually underestimates the home range size (Kochanny et al. 2009; Skupien et al. 2016). Much higher HR sizes (over 500 ha in summer and 300 ha in winter) were reported by Carvalho et al. (2008), who calculated HR as MCP95, but these studies described roe deer in mountain habitats after reintroduction. High HR sizes were also reported in the forest steppe landscape (Danilkin 1996). Most other studies reported much lower HRs, especially if the forest cover dominated the study site (e.g., Chapman et al. 1993; Fruziński et al. 1983; Lovari et al. 2017; Wasilewski 2001). Previous studies have indicated that forest cover has a significant effect on home range size (e.g., Lovari et al. 2017; Saïd and Servanty 2005; Tufto et al. 1996), a finding that was also confirmed by our research. Similar to our study, most studies reported larger individual home ranges for males than for females, although some reported the opposite (e.g., Cederlund 1983; Tufto et al. 1996; Vincent et al. 1995).

Previous studies have also shown that human activity modifies the HR and habitat utilization by roe deer, including hunting, recreational activities, or winter feeding (e.g., Bonnot et al. 2013; Guillet et al. 1996; Martin et al. 2018; Mols et al. 2022; Padié et al. 2015). As demonstrated, the movement patterns of European roe deer depended on roads and settlements, which could shape habitat utilization of this species, especially during the day (Bonnot et al. 2013; Coulon et al. 2008). However, no clear impact of anthropogenic infrastructure on roe deer HR size has been previously demonstrated, even if such an impact has been reported for other cervids (e.g., Jerina 2012). Saïd and Servanty (2005) initially included roads in the HR analysis but ultimately chose other environmental variables with greater importance. Finally, anthropogenic landscape features were not typically considered in HR size analyses (e.g., Bevanda et al. 2015; Saïd et al. 2009). We did not find a relationship between HR and settlement or roads in our study, but we did find a relationship between HR size and the impact of wind farms. Since this is a newly discovered phenomenon, it warrants discussion. For both HR metrics (MCP95 and KDE95), the number of turbines in the vicinity of roe deer significantly influenced HR size. This represents a new anthropogenic factor shaping roe deer's spatial behavior in agricultural landscapes. It is worth noting that turbines did not affect the size of the CA, only its location, with roe deer treating wind turbines similarly to buildings, as indicated by the lack of statistical difference in the distance of the CA from these two landscape elements.

Our results do not allow us to pinpoint the root causes of the wind turbine effect, that is, whether the infrastructure of the tall towers themselves, the noise and shadow flicker associated with rotating blades, or the increased presence of people near this infrastructure caused changes in habitat utilization (Gmaj and Klich 2026; Klich et al. 2020; Łopucki et al. 2017, 2018). It is also difficult to assess whether European roe deer perceive the surroundings of turbines as a landscape of fear. There are a few examples of increased vigilance at wind farms (e.g., Rabin et al. 2006), but such studies have not yet been conducted on roe deer. Cervids tend to reduce movement in the face of risk (e.g., Böhme et al. 2025; Little et al. 2016), and in such a case, we would observe lower daily distance traveled with an increase of wind farm impact on roe deer. Although daily distance traveled was not significantly correlated with wind farm impact, it showed an increasing trend, which may indicate occasional fleeing behavior. Similar conclusions can be drawn from the stronger effect of turbines on MCP95 size than on KDE95. The minimum convex polygon method is more sensitive to rare, long‐distance, or peripheral movements, which may reflect disturbance‐driven movements. The lack of a clear change in daily distance traveled may also indicate individual variability in roe deer's responses to wind turbines. Such differences were observed when examining the summer distribution of roe deer within a wind farm (Gmaj and Klich 2026). The authors showed that although some roe deer were located relatively close to the turbines, many others showed high clustering farther from this infrastructure. The “deterrent” effect of turbines results in roe deer avoiding them at an appropriate distance, traveling to or using foraging locations. This may be caused by the presence of people near the turbines, for example, service personnel, but also by high noise levels with variable parameters and rotating rotor blades. Locations of the studied animals indicate that roe deer usually do not pass in the immediate vicinity of the turbines, as evidenced by the example of a 2‐year‐old female with one of the turbines located in the central part of the HR (Figure 5). The passage of this individual to the south of the area was associated with avoiding the mentioned turbine.

**FIGURE 5:**
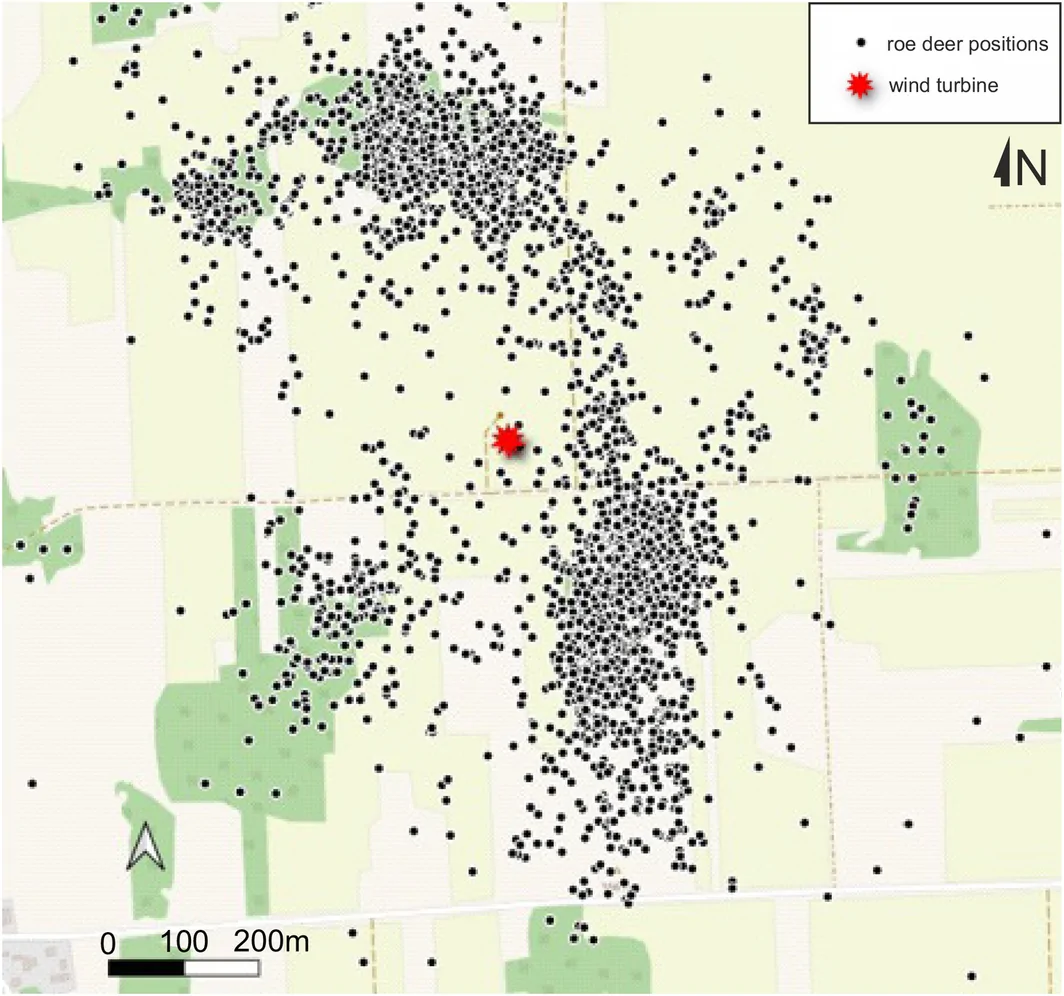
Locations of a 2‐year‐old female during winter in the central part of the HR. The GPS points show the roe deer bypassing the wind turbine as it moves between areas of use.

Evidence from cervids suggests that responses to anthropogenic stimuli are highly context‐dependent (e.g., Brown et al. 2012; Lynch et al. 2015). Studies on sika deer (Mai et al. 2026) indicate that human‐ or traffic‐related noise cues increase the probability of flight, whereas other disturbances primarily elevate vigilance or reduce foraging without causing displacement. Similar findings were reported for roe deer and red deer, but the responses differed between the species (Reimoser 2014). Moreover, behavioral responses vary with disturbance type and with intrinsic traits such as sex, age, reproductive status, and group size (Mai et al. 2026). In this context, the response of roe deer observed in our study appears relatively moderate. Although turbines were associated with larger home ranges, we did not detect clear effects on daily distance traveled, suggesting that individuals do not consistently respond with increased movement or frequent escape behavior. Roe deer in our study did not completely avoid turbine areas, and their core areas remained at measurable distances from this infrastructure, though increased. A slightly different effect was found for reindeer in relation to wind farms. Although home range sizes were not compared, shifts in home ranges after the construction of wind farms and changes in habitat use associated with this infrastructure were demonstrated (Skarin et al. 2018; Skarin and Alam 2017).

However, we would like to point out the limitations of our study. The population sample was relatively small, and although the results were generally consistent with previously demonstrated relationships, they should be considered a contribution to further research rather than a general description of the phenomenon. The study was also conducted mainly on younger individuals, which could have influenced the results, as such individuals are more prone to unpredictable behavior and movement. Furthermore, the study was based on a comparison of the intensity of wind farm impacts (number of turbines) rather than a typical BACI (Before‐After Control‐Impact) comparison, which would most accurately indicate changes in roe deer home range size.

The results, however, indicate that roe deer have probably adapted behaviorally to wind farms as a new landscape feature. They probably treat wind farms like human settlements, generally avoid them, and use habitats at an appropriate distance from the turbines. The comparable distance of the core area to wind turbines to that of settlements partially confirms this approach. The behavioral adjustment enables roe deer to reduce their physiological response to turbine‐related disturbances. Higher levels of stress hormones in roe deer were observed at larger wind farms, suggesting a limited potential for behavioral adjustment (Klich et al. 2020). However, this behavioral response comes at the expense of a larger home range, which implies higher energetic costs, which, especially during harsh winters, may result in lower body condition and reduced chances of survival or reproductive success (Lima 1998). However, these are only speculations that should be confirmed or refuted by other studies. Further research should also include a more detailed assessment of the behavior of roe deer and other terrestrial mammal species under the influence of wind farms. This will allow for a more comprehensive assessment of the impact of wind energy on terrestrial animals, which is still not possible today.

## Author Contributions

**Daniel Klich:** conceptualization (lead), data curation (lead), formal analysis (equal), funding acquisition (lead), investigation (lead), methodology (lead), resources (lead), writing – original draft (equal). **Michalina Gmaj:** data curation (supporting), formal analysis (equal), investigation (lead), methodology (supporting), writing – original draft (equal). **Wojciech Bielecki:** investigation (supporting), methodology (supporting), writing – review and editing (equal). **Maria Sobczuk:** investigation (supporting), writing – review and editing (equal). **Łukasz Mrugała:** investigation (equal), writing – review and editing (equal). **Paweł Nasiadka:** investigation (supporting), writing – review and editing (supporting). **Marta Gałązka:** investigation (equal), writing – review and editing (equal). **Marcin Świątek:** conceptualization (supporting), investigation (lead), methodology (supporting), writing – review and editing (lead).

## Funding

Funding for this research was provided by Narodowe Centrum Nauki in Poland (grant number: 2021/41/B/NZ9/04442).

## Conflicts of Interest

The authors declare no conflicts of interest.

## Supporting information


**Appendix S1:** Sizes of home ranges (MCP95, KDE95) and core areas (KDE50) and daily distances (DD) of roe deer in the agricultural landscape of central Poland in 2023–2026. Home ranges are expressed in hectares and daily distances in kilometers.

## Data Availability

The data that support the findings of this study are openly available in Appendix S1.
