# Supplementary material for: Roe Deer Capreolus capreolus Home Range Size and Daily Distance in the Agricultural Landscape of East‐Central Poland in Relation to Natural and Anthropogenic Factors
Source: Ecol Evol. 2026 Oct 28;16(11):e74550. doi: 10.1002/ece3.74550 (PMC13714632; doi:10.1002/ece3.74550)
Supplement: Supplementary file 1 [file ECE3-16-e74550-s001.pdf]

Appendix 1

Sizes of home ranges (MCP95, KDE95) and core areas (KDE50) and daily distances (DD) of roe deer in the agricultural landscape of central Poland in 2023-2026. Home ranges are expressed in hectares and daily distances in kilometers.

ID	Sex	Period	Year	MCP95	KDE95	KDE50	DD
SARN01	M	growing	2023	109.5	58.8	10.2	2.7
SARN01	M	winter	2023	56.8	47.6	8.2	3.4
SARN01	M	winter	2023/2024	107.9	80.8	12.8	3.7
SARN02	M	growing	2023	122.2	82.9	18.4	3.1
SARN02	M	winter	2023	108.4	67.9	11.0	3.7
SARN02	M	winter	2023/2024	164.1	80.2	12.5	3.4
SARN03	F	growing	2023	27.0	26.6	4.6	2.3
SARN03	F	winter	2023	50.7	44.2	7.2	3.3
SARN04	F	growing	2023	63.5	55.1	8.0	2.9
SARN04	F	winter	2023	104.8	67.4	12.5	3.8
SARN05	F	growing	2023	48.0	29.3	5.6	2.4
SARN05	F	winter	2023/2024	55.5	43.8	7.1	2.7
SARN06	F	growing	2023	91.5	41.7	5.9	2.6
SARN06	F	growing	2024	30.7	29.2	7.8	NA
SARN06	F	winter	2023/2024	650.3	93.0	10.4	2.9
SARN07	F	growing	2023	87.9	70.1	15.3	2.7
SARN07	F	winter	2023/2024	101.3	75.3	7.3	3.2
SARN17	F	growing	2025	112.8	79.6	13.6	2.9
SARN17	F	winter	2025	210.1	133.0	22.1	3.2
SARN18	F	winter	2025/2026	387.3	139.4	15.7	3.8
SARN21	M	winter	2025	168.9	103.1	12.9	4.3
